# Supplementary material for: Age and origin of a Cahokian wooden monument at the Mitchell site, Illinois, USA
Source: PLoS One. 2025 Oct 3;20(10):e0333783. doi: 10.1371/journal.pone.0333783 (PMC12494245; doi:10.1371/journal.pone.0333783)
Supplement: S1 — (DOCX) [file pone.0333783.s001.docx]

**S1. Supporting Data**


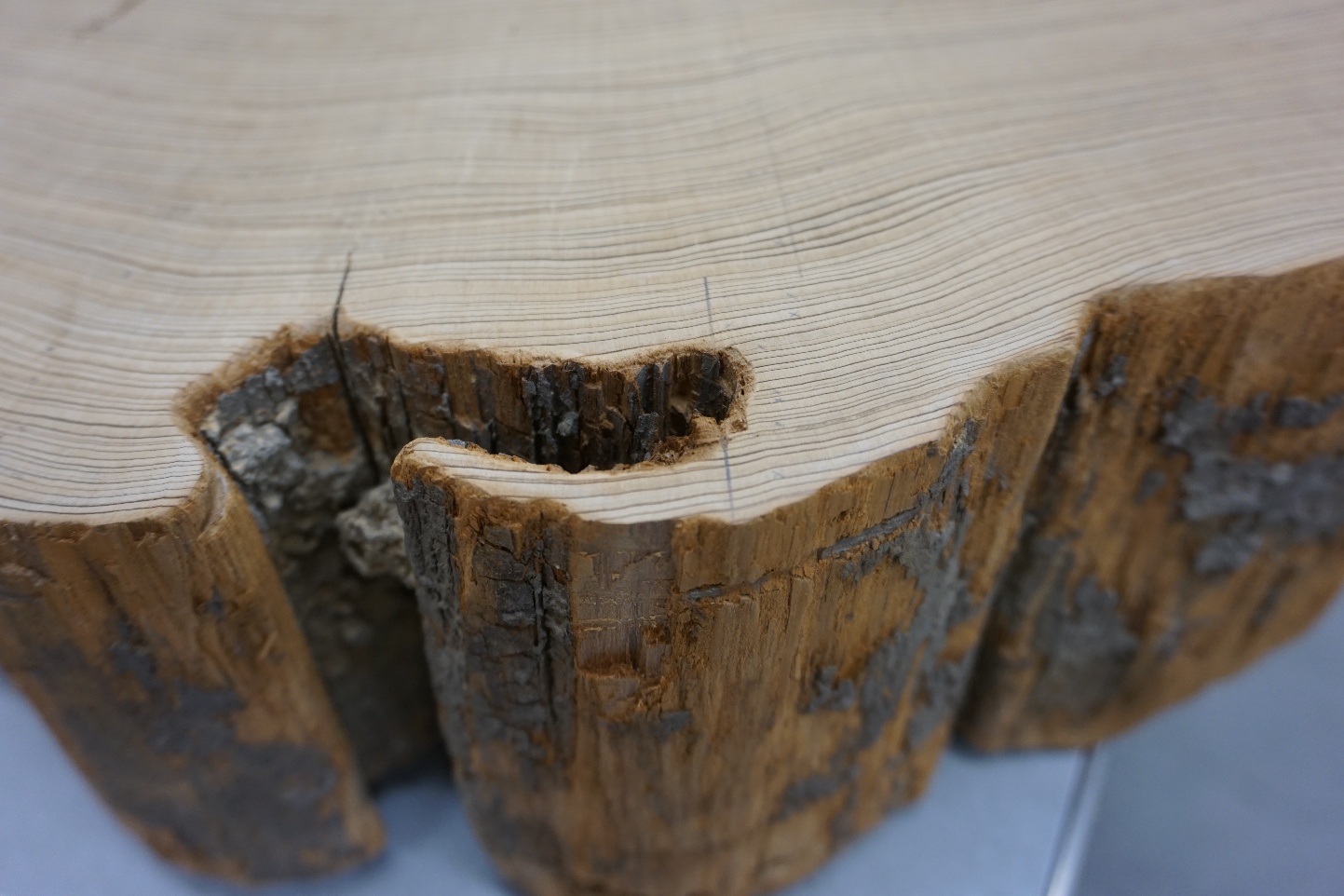


**Figure 1. Outer rings.** Photograph of the outermost rings of the Mitchell Log.

**Table 1. Low resolution C-14 data.** Summary of ^14^C measurements obtained in previous dating efforts.

| Lab# | sampled ring # | 14C age | ± |
| --- | --- | --- | --- |
| WIS-229 | 1-3 | 1110 | 55 |
| WIS-305 | 100-130 | 1000 | 150 |
| WIS-220 | 248-250 | 900 | 55 |

**Table 2. Low resolution C-14 data.** Summary of ^14^C measurements used to date the Mitchell Log in the initial low resolution wiggle-match calibration model.

| User# | AA# | sampled ring # | 14C age | ± |
| --- | --- | --- | --- | --- |
| Ms30-50-32 | AA115926 | 6 | 1149 | 23 |
| Ms30-50-33 | AA115928 | 46, 47, 48 | 1128 | 18 |
| mx50-30-9 | AA115571 | 56, 57, 58 | 1083 | 23 |
| Ms50-30-4 | AA115566 | 72 | 1046 | 21 |
| Ms50-30-5 | AA115567 | 96,97,98 | 996 | 21 |
| Ms50-30-6 | AA115568 | 134, 135, 136, 137, 138 | 895 | 21 |
| Mx30-50-34 | AA115930 | 193 | 974 | 18 |

**Table 3. Annual resolution C-14 data.** Summary of ^14^C measurements obtained to refine the date of the Mitchell Log by wiggle-matching to the 993/4 SEP.

| User# | AA# | sampled ring # | ^14^C age (yr) | ± (yr) |
| --- | --- | --- | --- | --- |
| Ms30-50-15 | AA115938 | 61 | 1071 | 24 |
| Ms30-50-16 | AA115939 | 62 | 1052 | 25 |
| Ms30-50-16 | AA115939r | 62 | 1,151 | 23 |
| Ms30-50-17 | AA115940 | 63 | 1088 | 24 |
| Ms30-50-17 | AA115940r | 63 | 1,093 | 23 |
| Ms30-50-18 | AA115941 | 64 | 1051 | 27 |
| Ms30-50-18 | AA115941r | 64 | 1,091 | 24 |
| Ms30-50-19 | AA115942 | 65 | 1018 | 24 |
| Ms30-50-19 | AA115942r | 65 | 1,068 | 24 |
| Ms30-50-20 | AA115943 | 66 | 1020 | 24 |
| Ms30-50-21 | AA115944 | 67 | 1023 | 24 |
| Ms30-50-22 | AA115945 | 68 | 1063 | 25 |
| Ms30-50-23 | AA115946 | 69 | 1011 | 23 |
| Ms30-50-24 | AA115947 | 70 | 1016 | 25 |
| Ms30-50-24 | AA115947r | 70 | 1,105 | 23 |
