# Supplementary material for: Age and origin of a Cahokian wooden monument at the Mitchell site, Illinois, USA
Source: PLoS One. 2025 Oct 3;20(10):e0333783. doi: 10.1371/journal.pone.0333783 (PMC12494245; doi:10.1371/journal.pone.0333783)
Supplement: S2 — (DOCX) [file pone.0333783.s002.docx]

**S2 D_Sequence model for the initial ^14^C dating of the Mitchell Log**

Plot()

{

D_Sequence("Mitchell Log")

{

R_Date("AA115926 ring 6",1149,23);

Gap(41);

R_Date("AA115928 ring 47",1128,18);

Gap(10);

R_Date("AA115571 ring 57",1083,23);

Gap(15);

R_Date("AA115566 ring 72",1046,21);

Gap(25);

R_Date("AA115567 ring 97",996,21);

Gap(39);

R_Date("AA115568 ring 136",895,21);

Gap(57);

R_Date("AA115930 ring 193",974,18);

Gap(1);

Date("Outermost ring");

};

};
