# Supplementary material for: Age and origin of a Cahokian wooden monument at the Mitchell site, Illinois, USA
Source: PLoS One. 2025 Oct 3;20(10):e0333783. doi: 10.1371/journal.pone.0333783 (PMC12494245; doi:10.1371/journal.pone.0333783)
Supplement: S3 — (DOCX) [file pone.0333783.s003.docx]

**S3. R script for reproducing annual tree-ring-radiocarbon calibration**

# load measurements and reference data

dr<- read.csv("~/projects/Greater Cahokia/reference_values_update.csv")

dm<- read.csv("~/projects/Greater Cahokia/measurement_values.csv")

dr$R_Date_1sigma[is.na(dr$R_Date_1sigma)]<- max(dr$R_Date_1sigma,na.rm=T)

dr<- dr[dr$year>=980 & dr$year <= 1010,]

# Reference values

RCr<- approx(x=dr$year,y=dr$R_Date,xout=unique(dr$year))

RCs<- approx(x=dr$year,y=dr$R_Date_1sigma,xout=unique(dr$year))

# Measurement values

x<- dm[c(1,5:6)]

# run Chi-squared test on all possible combinations

X2<- list()

for(i in 1:(length(RCr$x)-9)){

c<- RCr$y[i:(i+nrow(x)-1)]

sc<- RCs$y[i:(i+nrow(x)-1)]

r<- x$X14C.age

sr<- x$X14C.age.sigma

#X2[i]<- as.numeric(chisq.test(c,r,simulate.p.value = T)[1])

X2[i]<- sum(((r-c)^2)/((sr^2)+(sc^2)))

}

# cbind chi-squared test statistics and years

sig<- cbind(RCr$x[1:(length(RCr$x)-9)],unlist(X2))

# isolate the best match based on Chi-squared test

yrbest<- sig[which(sig[,2]==min(sig[,2],na.rm=T)),1]:

(sig[which(sig[,2]==min(sig[,2],na.rm=T)),1]+9)

# convert years to outermost ring age

sig[,1]<- sig[,1]+133

# plot it

layout(mat=matrix(c(1,2),1,2))

par(mar=c(4,4,1,1))

plot(x=dr$year,y=dr$R_Date,ylim=c(930,1200),pch=16,col=rgb(0,0,1,0.1),

ylab="14C age (yr B.P.)",xlab="Year (C.E.)")

rect(par("usr")[1],par("usr")[3],par("usr")[2],par("usr")[4],

col = "ghostwhite",border=NULL)

points(x=dr$year,y=dr$R_Date,pch=16,col=rgb(0,0,1,0.1))

abline(v=RCr$x,col='lightgray',lty='dotted')

abline(v=994,lty='dashed')

arrows(x0=dr$year,x1=dr$year,y0=dr$R_Date,

y1=dr$R_Date+dr$R_Date_1sigma,angle=90,length=0.02,col=rgb(0,0,1,0.1))

arrows(x0=dr$year,x1=dr$year,y0=dr$R_Date,

y1=dr$R_Date-dr$R_Date_1sigma,angle=90,length=0.02,col=rgb(0,0,1,0.1))

lines(RCr,col=rgb(0,0,1,0.4),lwd=2)

points(x=yrbest,y=x[,2],pch=8,cex=0.9,

col="red")

arrows(x0=yrbest,x1=yrbest,y0=x[,2],

y1=x[,2]+x[,3],angle=90,length=0.02,col="red")

arrows(x0=yrbest,x1=yrbest,y0=x[,2],

y1=x[,2]-x[,3],angle=90,length=0.02,col="red")

plot(sig,type='b',ylim=c(0,70),xlab="Final year (C.E.) of growth",

ylab="Chi-squared statistic (df = 10)")

rect(par("usr")[1],par("usr")[3],par("usr")[2],par("usr")[4],

col = "ghostwhite",border=NULL)

abline(v=sig[,1],col='lightgray',lty='dotted')

points(x=sig[,1],y=sig[,2],pch=16,cex=.5)

lines(x=sig[,1],y=sig[,2])

abline(h=qchisq(0.05,10,lower.tail=F),lty="dashed") # 95% upper limit

abline(v=RCr$x,col='lightgray',lty='dotted')
