# Supplementary material for: Age and origin of a Cahokian wooden monument at the Mitchell site, Illinois, USA
Source: PLoS One. 2025 Oct 3;20(10):e0333783. doi: 10.1371/journal.pone.0333783 (PMC12494245; doi:10.1371/journal.pone.0333783)
Supplement: S4 — (DOCX) [file pone.0333783.s004.docx]

S4. R script for Sr source analysis

library(assignR)

library(raster)

library(sp)

library(XML)

library(sf)

library(terra)

setwd("~/projects/Sr project/GIS")

shape<- st_read("states.shp")

cypressPoly<- st_read("cypress_poly.shp")

sites<- st_read("sites.shp")

mask<- shape

cypressGrid<- raster("cypress.tif")

cypressGrid<- projectRaster(cypressGrid,crs=crs(shape))

#writeRaster(cypressGrid,filename="cypressGrid",format="GTiff",overwrite=TRUE)

cypressPts<- rasterize(mask,cypressGrid)

cypressPts<- projectRaster(cypressPts,crs=crs(shape))

res(cypressPts)<- res(cypressGrid)

values(cypressPts)[values(cypressPts)>0]<- 1

values(cypressGrid)[values(cypressGrid)>0]<- 1

cypressGrid<- merge(cypressPts,cypressGrid)

plot(cypressGrid)

plot(mask,add=T)

plot(shape,add=T)

#writeRaster(cypressGrid,filename="cypressGrid",format="GTiff",overwrite=TRUE)

data<- getIsoscapes(isoType = "GlobalSr", timeout = 1200)

data

data_sr<- raster(data$sr_bio)

data_se<- raster(data$sr_bio_se)

data_sr<- projectRaster(data_sr,crs=crs(shape))

data_se<- projectRaster(data_se,crs=crs(shape))

data_sr<- crop(data_sr,shape)

data_se<- crop(data_se,shape)

plot(data_sr)

plot(shape,add=T)

cypress<- resample(x=cypressGrid,y=data_sr)

values(cypress)<- values(cypress)/sum(values(cypress),na.rm=T)

# produce coarse uncertainties following Bowen and Ma 2022 tutorial

# for Sr prediction

srmu<- data_sr

srsd<- data_se

#values(srsd)<- 0.001

srsurf<- stack(srmu,srsd)

# load Sr values for Mtichell and other locations

d<- read.csv("~/projects/Sr project/sr data.csv")

Dp<- data.frame("ID" = d[,1], "Sr" = d[,4])

pSurf<- pdRaster(srsurf,Dp,cypress)

plot(pSurf)

#alSurf<- pSurf[[1]]*pSurf[[2]]

#values(alSurf)<- values(alSurf)/sum(values(alSurf),na.rm=T)

#crSurf<- pSurf[[7]]*pSurf[[8]]

#values(crSurf)<- values(crSurf)/sum(values(crSurf),na.rm=T)

msSurf<- pSurf[[3]]*pSurf[[4]]

values(msSurf)<- values(msSurf)/sum(values(msSurf),na.rm=T)

#ksSurf<- pSurf[[5]]*pSurf[[6]]

#values(ksSurf)<- values(ksSurf)/sum(values(ksSurf),na.rm=T)

#alSurf<- aggregate(alSurf,5,fun="sum")

#alSurf<- mask(alSurf,mask)

#plot(alSurf,main="Allred Lake")

#plot(shape,add=T,)

#plot(sites[sites$name=="Allred Lake",],add=T,pch=16,cex=.4)

#writeRaster(alSurf, filename="alSurf", format="GTiff", overwrite=TRUE)

#crSurf<- aggregate(crSurf,5,fun="sum")

#crSurf<- mask(crSurf,mask)

#plot(crSurf,main="Cache River")

#plot(shape,add=T)

#plot(sites[sites$name=="Cache River",],add=T,pch=16,cex=0.4)

#writeRaster(crSurf, filename="crSurf", format="GTiff", overwrite=TRUE)

msSurf<- aggregate(msSurf,1,fun="sum")

msSurf<- mask(msSurf,mask)

plot(msSurf,main="Mitchell Site")

plot(shape,add=T)

plot(sites[sites$name=="Mitchell Site",],add=T,pch=16,cex=0.1)

writeRaster(msSurf, filename="msSurf.tiff", overwrite=TRUE)

#ksSurf<- aggregate(ksSurf,5,fun="sum")

#ksSurf<- mask(ksSurf,mask)

#plot(ksSurf,main="Kincaid Site")

#plot(shape,add=T)

#plot(sites[sites$name=="Kincaid",],add=T,pch=16,cex=0.1)

#writeRaster(ksSurf, filename="ksSurf", format="GTiff", overwrite=TRUE)

# odds ratio of different

getMeanIso<- function(x,m,n){

mras<- rasterize(m,x,fun='mean')

mras<- rasterToPoints(raster(mras))

ex<- mean(unlist(extract(x,mras[,1:2])),na.rm=T)

nulras<- rasterize(n,msSurf,fun='mean')

nulras<- rasterToPoints(raster(nulras))

nulex<- mean(unlist(extract(x,nulras[,1:2])),na.rm=T)

return(nulex/ex)

}

#odds ratios of Mitchell sample for different cypress stands

m1<- suppressWarnings(st_read(

list.files("~/projects/Sr project/GIS/stands")[1]))

mras<- rasterize(m1,msSurf,fun='mean')

mras<- rasterToPoints(raster(mras))

ex<- mean(unlist(extract(msSurf,mras[,1:2])),na.rm=T)

nulras<- rasterize(m1,msSurf,fun='mean')

nulras<- rasterToPoints(raster(nulras))

nulex<- mean(unlist(extract(msSurf,nulras[,1:2])),na.rm=T)

cypras<- rasterize(shape,msSurf)

cyppoints<- rasterToPoints(raster(cypras))

cypex<- extract(msSurf,cyppoints[,1:2])

dt<- data.frame(lon=cyppoints[,2],lat=cyppoints[,1],val=cypex/nulex)

coordinates(dt) <- ~ lon + lat # Convert data frame to spatial object

probs<- SpatialPointsDataFrame(cyppoints,

data=cypex/nulex)

odgrid<- rasterize(probs@coords,msSurf,probs@data)

plot(odgrid)

writeRaster(raster(odgrid), filename="msORBig", format="GTiff", overwrite=TRUE)

clipgrid<- mask(odgrid,cypressPoly)

plot(clipgrid)

writeRaster(raster(clipgrid), filename="msORClipped", format="GTiff", overwrite=TRUE)
