# Supplementary material for: Age and origin of a Cahokian wooden monument at the Mitchell site, Illinois, USA
Source: PLoS One. 2025 Oct 3;20(10):e0333783. doi: 10.1371/journal.pone.0333783 (PMC12494245; doi:10.1371/journal.pone.0333783)
Supplement: Attachment — Submitted filename: Response to Reviewers.docx [file pone.0333783.s006.docx]

Nicholas V. Kessler

Laboratory of Tree-Ring Research and AMS Laboratory

University of Arizona

Bannister Building, 1215 E Lowell St.

Tucson, AZ 85721

785-760-1730

Editor

PLOS One

Dear, Editor and Reviewers

We thank the editor and anonymous reviewer for their comments and suggestions. The critical point highlighted by the reviewer is an issue that the authors have considered carefully; prior to, during, and after writing. It is our view that a discussion of post extraction should remain in the article because it is of critical to contextualizing the historical importance of the post-pit phenomenon in the past. We have, in response to the concerns of the reviewer, revised our discussion of the timing of post extraction to emphasize that (1) we are not predicting an extraction date with the same resolution as our observed cutting date and that (2) the proposed timing of extraction is supported by independent information from other archaeological contexts and informs archaeological models for the process of the dissolution of the Cahokia phenomenon.

To address concerns about the hypothetical relationship between the proposed date of the poles extraction and its observed cutting date, we expand the explanation of the logic of our inferred indirect date for the pole extraction – that is based on the fact that when it was extracted the pole must have been weakened by rot at the ground surface sufficiently to break as it did. We re-phrase our discussion of this to clarify that this is an inference based on contextual clues from surrounding archaeological features and is copiously supported by references cited in the text. While not a formal hypothesis or model, this inference can be refuted with further research and is valid in the context of the discussion of the results. Importantly, we acknowledge the potential error inherent in such a claim and do not attempt to extend the precision of the pole’s cutting date to the inferred extraction date.

We also address the argument that the large number of marker posts on the ancient Cahokian landscape diminish the value of the post under study by clarifying in the introduction that the importance of dating marker posts is to gain a better understanding of the dissolution of Cahokian urbanism (a phenomenon of world historical importance) through charting the tempo of ritual marker pole emplacement. While we cannot do that with our sample of one, the paper’s contribution is that it clearly shows how this research could be accomplished technically. Furthermore, we emphasize that this is the first such marker pole, and a major and prominent one at that, to ever be directly dated at high-precision and sourced chemically.

Regarding the use of copyrighted material in Figure 4; this figure is an original product of the author and does not use map layers from proprietary sources. Permission to use images in Figure 1 are now provided in the submission.

Sincerely,

Nicholas V. Kessler (corresponding author)
